# GVAF: generalized, flexible filtering software for annotated variant files

**DOI:** 10.1007/s13258-024-01580-0

**Published:** 2024-10-12

**Authors:** Sora Kim, Sungwon Jung

**Affiliations:** 1 Department of Genome Medicine and Science, Gachon University College of Medicine, 38-13 Dokjeom-ro 3 beon-gil, Namdong-gu, Incheon, 21565 Republic of Korea; 2 Gachon Institute of Genome Medicine and Science, Gachon University Gil Medical Center, Incheon, Republic of Korea

**Keywords:** Variant filtering, Variant annotation, Command-line software, Next generation sequencing

## Abstract

**Background:**

In the rapidly advancing field of genomics, many tools have been developed to interpret genetic variants using next-generation sequencing (NGS) data. However, these tools often produce annotated variant files in different formats, which require specific software or programming skills to filter and analyze.

**Objective:**

To provide a filtering tool that can be used with diverse variant annotation tools without requiring specific software or programming skills.

**Methods:**

We developed Germline Variant Annotation and Filtering (GVAF), a command-line software tool that can handle annotated variant files in any table-shaped format. GVAF offers powerful filtering operations without the need for additional software or programming expertise.

**Results:**

Built on the Java framework and bash scripts, it provides extensive features, including flexible filtering rules, recognition of genotype-related fields from variant call format (VCF) files, and customizable result generation. GVAF also integrates easily into existing data analysis pipelines. Compared to other tools, GVAF offers a broader range of functionalities, making it more flexible and intuitive for managing annotated variant files.

**Conclusion:**

This GVAF software and online manual is publicly available at https://www.sysbiolab.org/gvaf for academic users and is designed to streamline the variant interpretation process, aiding researchers in producing meaningful results.

## Introduction

Since the availability of NGS, vast amount of genetic information has been generated with variant information from wide range of scientific fields, ranging from disease cohorts (Cancer Genome Atlas et al. 2013; Consortium 2020) to general populations (Consortium et al. 2015; Kurki et al. 2023). The variant call format (VCF) (Danecek et al. 2011) has been widely used as a standard and basic file format to describe such variant information. To interpret variants for different purposes, many software tools have been developed for annotating or filtering variants. Variant annotation tools such as The Ensembl Variant Effect Predictor (VEP) (McLaren et al. 2016), SnpEff (Cingolani et al. 2012) and Annovar (Wang et al. 2010) have been developed to associate variants with information from diverse public variant-related databases (e.g., dbSNP(Wheeler et al. 2008), 1000 genomes (Genomes Project et al. 2012), ESP; exome sequencing project (Lek et al. 2016), ExAC; Exome Aggregation Consortium (Exome Variant Server. NHLBI Exome Sequencing Project (ESP), ClinVar (Landrum et al. 2020) and etc.) while using VCF as a standard input format. Regarding the variant filtering, multiple software tools such as SnpSift (Cingolani et al. 2012), VCFTools (Danecek et al. 2011), GEMINI (Paila et al. 2013), BCFTools (Danecek et al. 2021) and Slivar (Pedersen et al. 2021) have been developed to find variants of interests. Combined use of such tools can be necessary for efficient analysis of genomic variants. In general, different annotation tools generate different output files of annotated variants in their own formats with different information fields depending on their annotation sources. Informed filtering of annotated variants is often necessary for many studies with consideration of such annotation during the filtering process. However, many variant filtering tools consider only VCF files as input and rarely support annotated variant files. Some software tools require specific file formats for input or require extra software such as in-house code scripts to handle annotated variant files. To overcome such limitations, we developed a command-line software tool GVAF, which handles arbitrary table-shaped annotated variant files and provides powerful filtering operations without requiring extra software of programming skills.

## Materials and methods

**Fig. 1:**
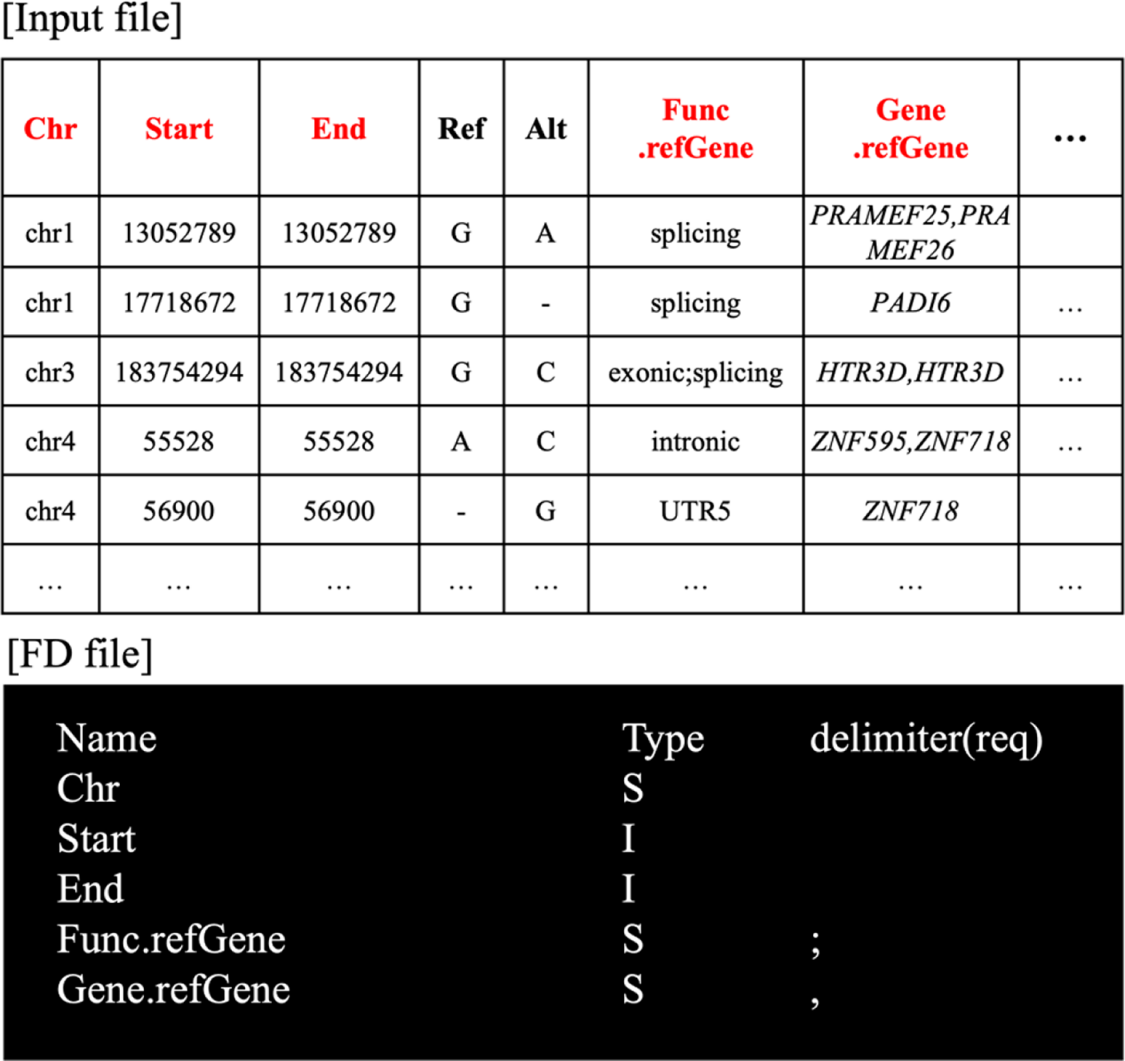
An example of input file configuration. Input files can be recognized by preparing an FD file. An FD file declares the fields (“Name”), types of fields (“Type”) and delimiters for the field values (“delimiter(req)”) to read specific fields from the table-shaped input file. Data type of a field can be defined using character codes such as S (string), I (integer) and D (real number). Delimiters can be defined for the fields that may have multiple values (Refer online documents for complete details.)

GVAF can process input text files in any tabular format by recognizing the fields of the table as defined by a user and applying various manipulative functionalities to the recognized fields and their values. Specifically, GVAF is primarily designed to recognize tab-separated text (TSV) files as input while using the concept of *FieldDefinition* (FD) to allow users to define the characteristics of fields in input files as shown in Fig. 1. By leveraging such flexible input field recognition, GVAF can provide various manipulation functions for the recognized fields to utilize type-specific operations while handling multi-valued fields.

**Fig. 2:**
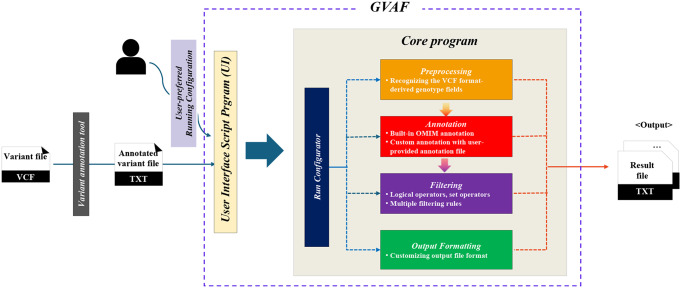
The architecture of GVAF. GVAF accepts text-format input files that are generated from conventional variant annotation tools along with user-provided options for running configurations. The user interface script program configures run-setting for the *Core program* and passes the input file. *Core program* runs necessary steps of *Preprocessing*, additional *Annotation*, *Filtering* and *Output Formatting* processes by following the run-setting, then generates output files in a user-defined format

**Fig. 3:**
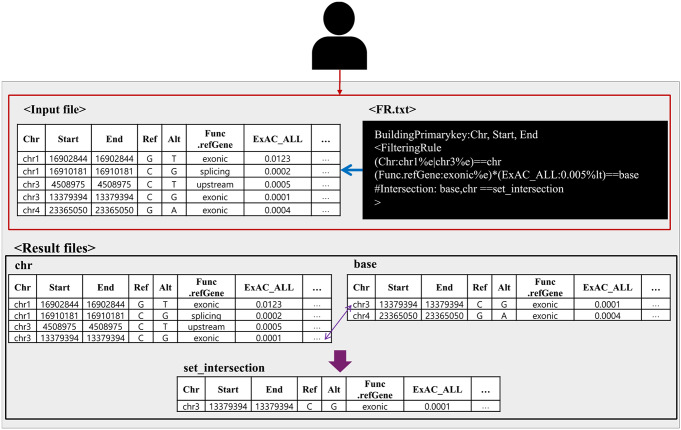
An example of how *Filtering* module works. Variant information in input files can be filtered and managed based on a user-defined filtering rule (FR) file. An FR file describes the way each variant is uniquely defined and filtering rules for variants. The fields that are specified by “BuildingPrimarykey” are combined to make the unique ID of a variant, and each line in “FilteringRule” area defines a filtering rule or a set operation on intermediate filtering results. The example FR file shows three filtering rules with names of “chr”, “base” and “set_intersection” as shown at the end of individual lines. “chr” and “base” are filtering rules with comparison (“%e”: equal to, “%lt”: less than) and logical (‘|’: OR, ‘*’: AND) operators on the specified fields (on the “Chr”, “Func.refGene” and “ExAC_ALL” fields) (see online GVAF documents for complete list of operations and examples). From this example, variants that are on chromosomes 1 or 3 are filtered by the rule “chr” and variants on exonic region with the reported frequency less than 0.005 from the ExAC database are filtered by the rule “base”, then their intersection is made by the set operation rule “set_intersection” (that starts with ‘#’ at the beginning of the line)

**Fig. 4:**
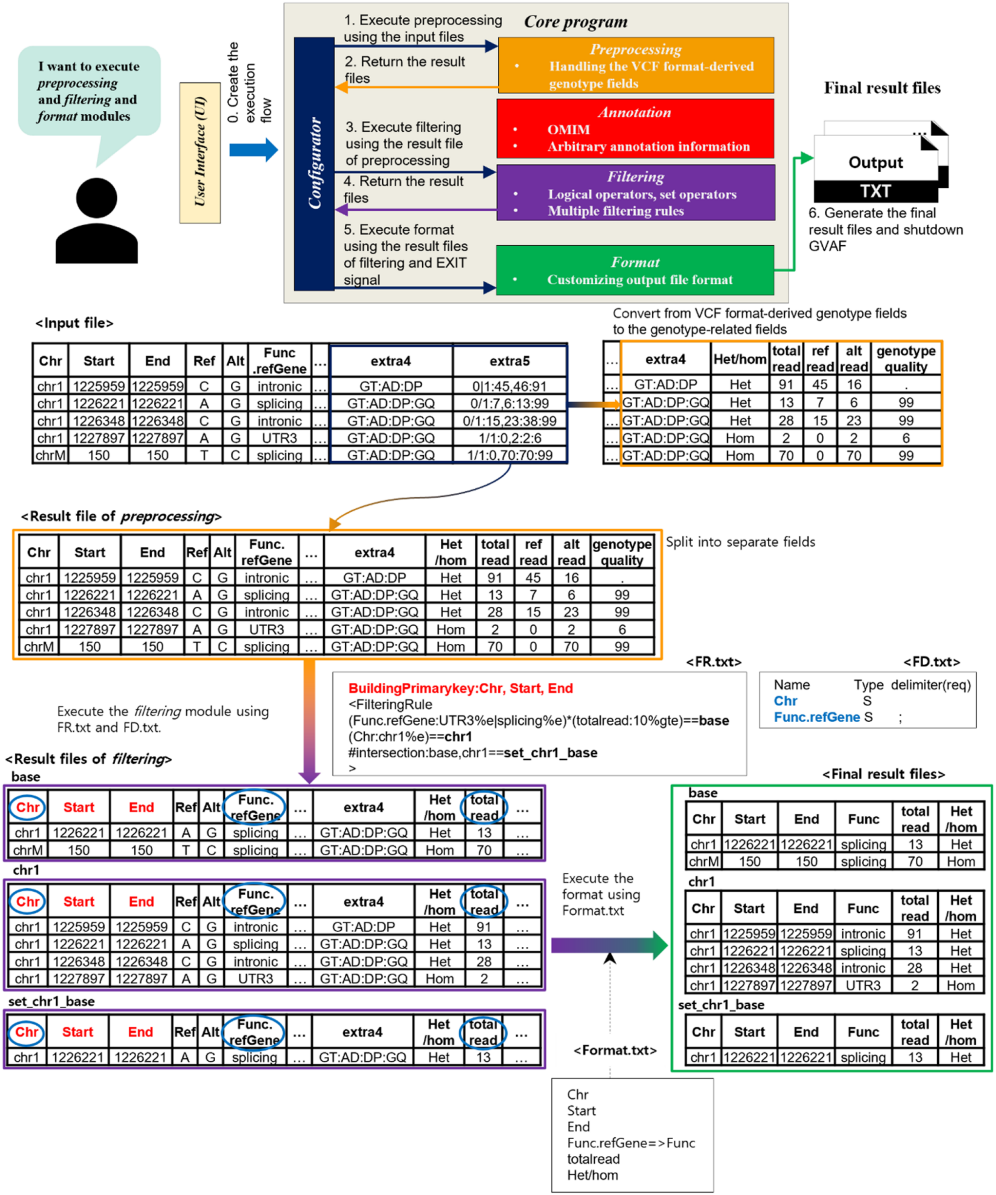
An example scenario of using GVAF. In this example, the input file has a format like the output of Annovar. The *Preprocessing* module creates an intermediate file by converting the VCF-derived genotype information into separate, user-friendly fields. The intermediate file from the *Preprocessing* module is used as input for the next module in the execution flow, the *Filtering* module. The *Filtering* module applies three filtering rules that are defined in the FR file (FR.txt) to the intermediate file from the *Preprocessing* module to generate filtering results, and the filtering result files are fed into the *Formatting* module to generate output files that follow the user-defined format. With user-supplied options to configure run configurations, GVAF can be used to perform arbitrary combination of different modules with flexible filtering rules and formatting functionality

We developed GVAF as a software running in a command-line environment to enable easy integration into existing software pipelines on Linux-like operating systems. Figure 2 shows the overall architecture of GVAF, where GVAF consists of two programs: *UI* (User Interface) written in bash script and the *Core program* written in JAVA. *UI* are designed to configure run-setting for *Core program* based on user-preferred options and pass input files. The *Core program* provides key manipulation functions for handling annotated variant information from input files. The *Core program* consists of five modules - *Configurator*,* Preprocessing*,* Annotation*,* Filtering* and *Formatting*. Each module provides different manipulation functionalities, while *Configurator* configures run-setting for GVAF and generates execution flow of necessary modules based on the user-provided options. The *Preprocessing* module explores the VCF-derived genotype information by recognizing relevant fields such as genotype and read counts, and each information can be treated as a separate field. The *Annotation* module adds additional annotation information to the already annotated variants using the built-in An Online Catalog of Human Genes and Genetic Disorders (OMIM) (Hamosh et al. 2002) information or user-supplied custom annotation information. The *Filtering* module selects variants of interest by applying user-defined filtering rules to variants and their annotated information, while multiple filtering rules can used at the same time (Fig. 3). Each filtering rule can be defined with combinations of comparison, logical, and set operators (union, difference, and intersection) along with brackets for further complex rule definitions (please refer the *Filtering* section of the software manual document for the complete description on the syntax and examples of defining filtering rules). Set operations between filtering results from multiple different input files are also supported. The *Formatting* module allows users to customize the output file format, where users can selectively export necessary fields and change their names. The *Formatting* module generates an output file for each filtering rule, thus allows users to run as many filtering tasks as necessary. An example scenario of using GVAF with relevant data transformation and transfer is shown in Fig. 4. Users can selectively use the whole or part of the four modules based on their needs.

## Results

To summarize the characteristics of GVAF in managing variant information, we compared its characteristics with that of multiple other command-line software tools (VCFTools, GEMINI and SnpSift) for similar purposes. Their characteristics in comparison are shown in Tables 1 and 2.

Table 1 shows the comparison of key features regarding user friendliness and the flexibility of usage between GVAF and other software tools, and it clearly demonstrates the benefit of using GVAF. Table 2 showcases the powerful filtering functionalities of GVAF for handling variant annotation information. Thus, GVAF provides superior functionality for handling annotated variant information by offering extensive functionalities in user-friendly manner while supporting arbitrary tab-delimited formats from diverse variant annotation tools. As it has been developed to minimize the need for additional burden on preparing computing environments (such as the installation of pre-requisite programs or preparing input with specific formats) as well as getting familiar with programming skills for filtering operations, GVAF can be also easily utilized in various computing environments by users even with limited knowledge on computer skills.

**Table 1:** Comparison of key features between GVAF and the other software tools

	VCFTools	GEMINI	SnpSift	BCFTools	Slivar	GVAF
Input file format	vcf/bcf	vcf	vcf	vcf/bcf	vcf	tsv^*^
No extra software required	X	X	O	O	O	O
No programming skill required for filtering	O	O	X	X	X	O
Supporting multiple files as input	X	X	X	X	X	O
Application of multiple filtering criteria in a single execution	X	X	X	X	X	O
Supporting multicore processors	X	O	X	O	O	O
Batch run of selected sub-tasks	X	O	X	X	X	O

*: Tab-delimited text file format

X: Not supported

O: Supported

**Table 2:** Comparison of filtering functionalities between GVAF and the other software tools

	VCFTools	Gemini	SnpSift	BCFTools	Slivar	GVAF
Logical operators available	O	O	O	O	O	O
Comparison operators available	O	∆^*^	O	O	O	O
Searching keywords with exact match	O	∆^*^	O	O	O	O
Searching keywords with partial match	X	∆^*^	X	O	O	O
Comparison operators available between different filtering results	X	X	X	X	X	O

X: Not supported

O: Supported

∆: Partially supported

^*^: GEMINI recognizes annotated fields only from result of SnpEff or VEP

## Discussion

We developed a software tool GVAF that provides extensive manipulation functions with high flexibility for handling annotated variant information, and it allows users to apply multiple filtering operations at the same time to the input in user-defined format. It also allows users to supple the variant information with additional custom annotations, and its filtering rules of varying complexity can be defined by users using written expressions without requiring programming skills. GVAF consists of multiple processing modules to support user-specific task requirements, where all or part of the processing modules can be selectively used by command-line options with ease. Considering that there has been limited availability of user-friendly software to handle annotated variant information, GVAF can provide various benefits to wide range of users with its user-friendliness, easy adaptability to variant processing pipelines, and powerful filtering functionality. As more genetic studies for large scale cohorts emerge, software tools such as GVAF can be great help to users who need to process vast amount of annotated variant information.

## Data Availability

Executable program files and example data for GVAF can be downloaded at https://www.sysbiolab.org/gvaf, which also provides installation instructions and the manual documentation. Detailed descriptions with various execution examples are provided in the manual documentation.
